# Exploring the Multi-Faceted Potential of Carob (*Ceratonia siliqua* var. Rahma) Leaves from Morocco: A Comprehensive Analysis of Polyphenols Profile, Antimicrobial Activity, Cytotoxicity against Breast Cancer Cell Lines, and Genotoxicity

**DOI:** 10.3390/ph16060840

**Published:** 2023-06-05

**Authors:** Amine Elbouzidi, Mohamed Taibi, Hayat Ouassou, Sabir Ouahhoud, Douâae Ou-Yahia, El Hassania Loukili, Marouane Aherkou, Farid Mansouri, Noureddine Bencheikh, Salah Laaraj, Reda Bellaouchi, Ennouamane Saalaoui, Kaoutar Elfazazi, Abdelbasset Berrichi, Malika Abid, Mohamed Addi

**Affiliations:** 1 Laboratoire d’Amélioration des Productions Agricoles, Biotechnologie et Environnement (LAPABE), Faculté des Sciences, Université Mohammed Premier, Oujda 60000, Morocco; mohamedtaibi9@hotmail.fr (M.T.); f.mansouri@ump.ac.ma (F.M.); abdel20759@yahoo.fr (A.B.); m.abid@ump.ac.ma (M.A.); 2 Centre de l’Oriental des Sciences et Technologies de l’Eau et de l’Environnement (COSTEE), Université Mohammed Premier, Oujda 60000, Morocco; douaae.ouyahia@usmba.ac.ma (D.O.-Y.); e.loukili@ump.ac.ma (E.H.L.); 3 Higher Institute of Nursing Professions and Health Techniques, Oujda 60000, Morocco; hayatouassou@gmail.com; 4 Laboratory of Bioresources, Biotechnology, Ethnopharmacology and Health, Faculty of Sciences, Mohammed First University, Boulevard Mohamed VI, Oujda 60000, Morocco; s.ouahhoud@ump.ac.ma (S.O.); bencheikh_noureddine1718@ump.ac.ma (N.B.); r.bellaouchi@ump.ac.ma (R.B.); e.saalaoui@ump.ac.ma (E.S.); 5 Biotechnology Laboratory (MedBiotech), Bioinova Research Center, Rabat Medical and Pharmacy School, Mohammed Vth University, N.U, Rabat B.P 8007, Morocco; aherkou.marouane@gmail.com; 6 Centre Mohammed VI For Research and Innovation (CM6), Madinat Al Irfane, Rabat B.P 6212, Morocco; 7 Regional Center of Agricultural Research of Tadla, National Institute of Agricultural Research (INRA), Avenue Ennasr, Rabat Principal, Rabat 10090, Morocco; salah.laaraj@usms.ma (S.L.); kaoutar.elfazazi@inra.ma (K.E.)

**Keywords:** *Ceratonia siliqua* L., Morocco, HPLC-DAD, phenolics, antioxidant activity, antimicrobial activity, cytotoxicity, breast cancer, genotoxicity

## Abstract

The botanical species *Ceratonia siliqua* L., commonly referred to as the Carob tree, and locally as “L’Kharrûb”, holds significance as an agro-sylvo-pastoral species, and is traditionally utilized in Morocco for treating a variety of ailments. This current investigation aims to ascertain the antioxidant, antimicrobial, and cytotoxic properties of the ethanolic extract of *C. siliqua* leaves (CSEE). Initially, we analyzed the chemical composition of CSEE through high-performance liquid chromatography with Diode-Array Detection (HPLC-DAD). Subsequently, we conducted various assessments, including DPPH scavenging capacity, β-carotene bleaching assay, ABTS scavenging, and total antioxidant capacity assays to evaluate the antioxidant activity of the extract. In this study, we investigated the antimicrobial properties of CSEE against five bacterial strains (two gram-positive, *Staphylococcus aureus*, and *Enterococcus faecalis*; and three gram-negative bacteria, *Escherichia coli*, *Escherichia vekanda*, and *Pseudomonas aeruginosa*) and two fungi (*Candida albicans*, and *Geotrichum candidum*). Additionally, we evaluated the cytotoxicity of CSEE on three human breast cancer cell lines (MCF-7, MDA-MB-231, and MDA-MB-436) and assessed the potential genotoxicity of the extract using the comet assay. Through HPLC-DAD analysis, we determined that phenolic acids and flavonoids were the primary constituents of the CSEE extract. The results of the DPPH test indicated a potent scavenging capacity of the extract with an IC_50_ of 302.78 ± 7.55 µg/mL, which was comparable to that of ascorbic acid with an IC_50_ of 260.24 ± 6.45 µg/mL. Similarly, the β-carotene test demonstrated an IC_50_ of 352.06 ± 12.16 µg/mL, signifying the extract’s potential to inhibit oxidative damage. The ABTS assay revealed IC_50_ values of 48.13 ± 3.66 TE µmol/mL, indicating a strong ability of CSEE to scavenge ABTS radicals, and the TAC assay demonstrated an IC_50_ value of 165 ± 7.66 µg AAE/mg. The results suggest that the CSEE extract had potent antioxidant activity. Regarding its antimicrobial activity, the CSEE extract was effective against all five tested bacterial strains, indicating its broad-spectrum antibacterial properties. However, it only showed moderate activity against the two tested fungal strains, suggesting it may not be as effective against fungi. The CSEE exhibited a noteworthy dose-dependent inhibitory activity against all the tested tumor cell lines in vitro. The extract did not induce DNA damage at the concentrations of 6.25, 12.5, 25, and 50 µg/mL, as assessed by the comet assay. However, the 100 µg/mL concentration of CSEE resulted in a significant genotoxic effect compared to the negative control. A computational analysis was conducted to determine the physicochemical and pharmacokinetic characteristics of the constituent molecules present in the extract. The Prediction of Activity Spectra of Substances (PASS) test was employed to forecast the potential biological activities of these molecules. Additionally, the toxicity of the molecules was evaluated using the Protox II webserver.

## 1. Introduction

Globally, breast cancer (BC) represents a notable factor in women’s mortality rates, as evidenced by the staggering number of reported cases in 2018, which amounted to approximately 2.1 million. Additionally, the disease was responsible for approximately 630,000 deaths during the same period [1]. Despite considerable progress in early detection and advanced treatment modalities, BC remains a major global health challenge, largely due to the inherent genetic heterogeneity that underlies drug resistance. BC is a malignant neoplasm that originates from the epithelial tissue of the mammary gland and annually affects over 1.3 million women worldwide. Among its molecular subtypes, the triple-negative (TNBC) or positive (TPBC) have garnered considerable research attention. Experimental and clinical studies have demonstrated the crucial involvement of estrogen in the proliferation and development of BC, mediated by its interaction with specific estrogen receptors, alpha (ER-α) and beta (ER-β) [2].

The production of reactive oxygen species (ROS) constitutes a fundamental mechanism linked to the onset and advancement of breast cancer. As a consequence, there is an elevation in markers of oxidative stress and a reduction in the efficiency of antioxidant defense mechanisms [3,4]. ROS may also contribute to the early stages of cancer by promoting processes such as fibroblast proliferation and epithelial hyperplasia, which can alter the architecture of breast tissue. DNA analysis of breast cancer patients has revealed the presence of 8-hydroxydeoxyguanosine, which may be involved in the development of the disease. Current treatments for BC include surgery, hormone therapy, and chemo-radiotherapy. However, the severity, dosage, and duration of treatment depend on various factors, including tumor size, subtype, and staging [5,6]. Synthetic chemotherapeutic agents are used to combat cancer cells, but their non-specific effects on normal cells can cause adverse reactions [7]. Consequently, there is a growing need for novel, cost-effective, and efficacious anticancer therapies derived from natural and safer sources. Medicinal plant-based remedies have several advantages over synthetic chemical molecules, including reduced side effects, enhanced activity, decreased expenses, and broader accessibility. Plants have been utilized as sources of herbal medicine for human ailments since ancient times, and modern research has identified more than 3000 medicinal plant species that exhibit antitumor properties [1]. Thirty natural products derived from plants have undergone clinical evaluation for their anticancer properties. The use of natural products as alternative therapeutic agents for cancer and other medical conditions has gained popularity due to their proven therapeutic effectiveness and minimal toxicity [8,9,10].

The use of plant extracts as a potential treatment for pathogens and cancer is justified by their antimicrobial compounds and natural antioxidants that eliminate harmful free radicals. The medicinal properties of plants are known to prevent and reduce the negative effects of conventional treatments. Additionally, using plant extracts in combination with other therapies has promising potential to combat a range of microbes and serious diseases like cancer.

Carob (*Ceratonia siliqua* L.) is a valuable perennial tree originally from the Mediterranean region. It is extensively grown not only in the Mediterranean basin but also in other geographic regions sharing similar climatic characteristics [11]. This species is highly adaptable to harsh environmental conditions, including drought, salinity, and poor soils, and requires minimal cultural inputs [12,13]. The carob tree is highly valued for its economic and environmental benefits, and its fruit, known as a pod, is a valuable source of food and industrial raw materials [14,15,16]. The carob pod is a composite structure consisting of two primary constituents, namely the seeds and the pulp. The pulp, constituting about 90% of the pod, is a highly nourishing substance that is rich in a diverse range of biologically active compounds, including but not limited to polyphenols, sugars, cyclitols, amino acids, fibers, and minerals. On the other hand, carob seeds are equally valuable and comprise gum, polyphenols, and proteins. Despite the value of carob pulp and seeds, the leaves of the carob tree are often neglected. Carob leaves contain a range of biologically active compounds, including polyphenols, flavonoids, and tannins, and have been traditionally used for medicinal purposes [17,18,19,20,21]. However, there is limited research on the potential uses of carob leaves, and they are often considered a byproduct of carob production.

The present study presents a detailed account of the potential biological activities associated with the ethanolic extract of carob leaves. In line with this, the primary objective of this research was to scrutinize the chemical composition of the ethanolic extract of *C. siliqua* (CSEE) by employing HPLC-DAD and further assess its plausible applications in diverse biological domains. These included examining its antioxidant and antimicrobial properties, assessing its potential to inhibit the proliferation of three categories of breast cancer cells (namely, MCF-7, MDA-MB-231, and MDA-MB-436), and studying its genotoxicity. The physicochemical properties of each identified molecule in the extract, including drug-likeness and pharmacokinetic properties, were determined using computational analysis. A Prediction of Activity Spectra of Substances (PASS) test was conducted to predict the biological activities of the molecules, while their potential toxicity was assessed using the Pro-Tox II webserver. These results could provide valuable information regarding the potential therapeutic applications of CSEE as a natural product.

## 2. Results and Discussion

### 2.1. Phytochemical Analysis Using HPLC-DAD

**Figure 1:**
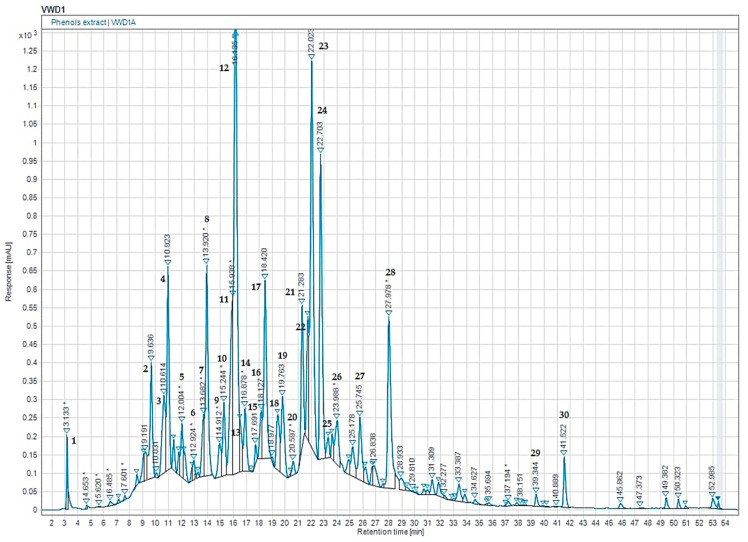
HPLC-DAD chromatogram of the phenolic composition of CSEE. Note: the corresponding molecules are in Table 1.

In our study, we utilized phytochemical analyses to evaluate the chemical composition of ethanolic extracts of carob using the HPLC-DAD technique (Figure 1 and Table 1). Each HPLC/UV profile was conducted at 280 nm, and the analysis detected 20 peaks. Our findings indicated that the main substances found in the extracts were naringin, succinic acid, 2-hydroxycinnamic acid, flavone, phloridzin dihydrate, 3-hydroxybenzoic acid, orcinol, and syringic acid. Naringin, which belongs to the flavanone’s derivatives, was the most prevalent compound at 17.54%, followed by succinic acid at 12.07%, and 2-hydroxycinnamic acid at 8.31%. Flavone was the most prominent compound detected for the flavonoid profile at 6.23%, followed by quercetin 3-*O*-β-D-glucoside at 2.58%. Our analysis also detected kaempferol in the extract at 1.65%, while chalcone was present in modest concentrations.

Several studies have identified the principal phenolic compounds present in carob extracts, but their levels vary. A previous study by Eldahshan (2010) [22] has reported the presence of gallic acid, quercetin 3-*O*-β-D-glucoside (isoquercetin), kaempferol 3-*O*-α-L-rhamnoside (afzelin), quercetin 3-*O*-α-L-rhamnoside (quercitrin), 1,2,6 tri-*O*-galloyl-β-D-glucopyranose, (-)-epigallocatechin-3-*O*-gallate, kaempferol, and quercetin in the ethanolic extract of carob leaves. Similar compounds have been previously reported in carob pods and derived products. For instance, Goulas et al. (2019) [23] found that gallic acid and rutin were the major phenolic compounds detected in carob powder. These findings suggest that carob extract could potentially have various therapeutic applications. The detection of naringin as the most abundant constituent in our extract is significant, given its link to various health advantages, such as anti-inflammatory and antioxidant effects [24,25]. Similarly, the detection of quercetin and kaempferol in our extract suggests that carob may also have potential anticancer properties, as these compounds have been shown to have anticancer effects [26,27,28,29,30,31].

### 2.2. TPC, TFC, and TCT Contents

The pharmacological activities of natural products are greatly influenced by polyphenols. Environmental factors including harvest season, extraction method, and storage conditions have a significant impact on their composition [32].

Several studies indicate that phenolic compounds have a significant impact on human health because of their ability to act as antioxidants [33]. The amounts of total phenolics, flavone, flavonol, and condensed tannins contents of ethanolic extract from *C. siliqua* (CSEE) are shown in Table 2. The total phenols of *C. siliqua* ethanolic extract was 96.98 ± 1.15 mg GAE/100 g DW. The flavonoids content was 5.92 ± 0.06 mg RE/100 g DW. The total condensed tannins of CSEE was 29.61 ± 0.36 mg CE/100 DW. The current study’s findings align with Gregoriou et al.’s (2021) research on polyphenol content in extracts from *C. siliqua* [34]. Additionally, Ydjedd et al. (2017) reported that the ethyl acetate extracts of *C. siliqua* had the highest levels of phenolic and flavonoid contents [35]. Moreover, Avallone et al.’s (1997) investigation revealed the notable presence of condensed tannins in carob [36].

### 2.3. Physiochemical and Pharmacokinetic Properties (ADME) of CSEE

Table 3 presents the physiochemical and drug-likeness analysis of 14 major compounds found in CSEE. The analysis includes parameters such as Hydrogen-Bond Donors (HBD), Hydrogen-Bond Acceptors (HBA), Topological Polar Surface Area (TPSA), distribution coefficient (Log Po/w), and solubility (Log S). Additionally, the Lipinski’s Rule of Five and a Veber filter were applied to assess drug-likeness. The results indicate that all 14 compounds have moderate to high solubility (+++ to +) and adhere to Lipinski’s Rule of Five and Veber filter requirements. Overall, the majority of the compounds exhibit good solubility and moderate to high BBB permeability, indicating that they can easily cross the blood-brain barrier. All but three compounds (naringin, salicylic acid, and phloridzin dihydrate) comply with Lipinski’s Rule of Five. These compounds have violations related to molecular weight, the number of oxygen atoms, and the number of hydrogen-bond donors, respectively. The compounds with the highest potential drug-likeness score are catechin **(2)**, 4-hydroxybenzoic acid **(3)**, orcinol **(4)**, quercetin **(9)**, and chalcone **(10)**. The results suggest that these compounds have potential therapeutic applications due to their physiochemical properties and adherence to drug-likeness criteria. Nonetheless, additional in vitro and in vivo investigations are imperative to verify their effectiveness and safety.

Table 4 presents the pharmacokinetic properties (ADME) of 14 of the most abundant compounds in CSEE extract. The parameters are categorized into four groups: absorption parameters, distribution parameters, metabolism parameters, and excretion parameters. The bioavailability score predicts the fraction of an orally administered compound that reaches systemic circulation. The bioavailability score ranges from 0 to 1, and a score closer to 1 indicates better bioavailability [37]. Among the identified compounds, it was found that 4-Hydroxybenzoic acid, 3-Hydroxybenzoic acid, Quercetin 3-*O*-β-D-glucoside, Quercetin, Chalcone, Succinic acid, 2-Hydroxycinnamic acid, and Flavone have good bioavailability scores (≥0.55). Caco-2 permeability predicts the intestinal permeability of a compound using the Caco-2 cell model [38]. The value of Caco-2 permeability ranges from −2 to 2, and a higher value indicates better intestinal permeability. Among the identified compounds, Orcinol, 3-Hydroxybenzoic acid, Quercetin 3-*O*-β-D-glucoside, Quercetin, Chalcone, Succinic acid, 2-hydroxycinnamic acid, Phloridzin dihydrate, and Flavone have good intestinal permeability (≥1.0). For the Intestinal Absorption, which is an important parameter that predicts the percentage of orally administered compound that is absorbed by the intestine, a higher value indicates better absorption. Among the identified compounds, Naringin, 4-Hydroxybenzoic acid, 3-Hydroxybenzoic acid, Quercetin 3-*O*-β-D-glucoside, Quercetin, Chalcone, Succinic acid, 2-hydroxycinnamic acid, Phloridzin dihydrate, and Flavone have good intestinal absorption (>70%).

The identified compounds were analyzed based on their distribution parameters. Log Kp (cm/s) was used to predict the ability of a compound to permeate biological membranes, and none of the identified compounds were found to have good membrane permeability. VDss was used to predict the volume of distribution of a compound in steady-state, and only Catechin and Salicylic acid showed good tissue distribution, with higher values indicating higher tissue distribution. The blood-brain barrier (BBB) permeability was utilized to anticipate the capacity of a compound to traverse this biological barrier; however, none of the identified compounds demonstrated favorable BBB permeability despite the BBB permeability value range of −3 to 3.

In order to anticipate the potential drug metabolism or toxicity of a molecule, it is important to examine its predicted activity and interactions with cytochrome P450 (CYP) isozymes. The activity of a molecule can refer to its effects on biological systems, such as its ability to bind to specific receptors or enzymes. Understanding how a molecule interacts with CYP isozymes, which are responsible for metabolizing many drugs, can provide important insights into its potential pharmacokinetic properties [7]. None of the identified compounds are substrates for the cytochrome P450 (CYP) enzymes CYP2D6 and CYP3A4, which are responsible for metabolizing many drugs [7]. Similarly, none of the identified compounds are inhibitors of the activity of CYP2D6 and CYP3A4 enzymes. The Renal Organic Cation Transporter 2 (OCT2) plays a key role in removing various drugs from the body through the kidneys. When a drug is a substrate for OCT2, it can be excreted more quickly through urine. Efficient renal clearance, facilitated by major organic cation transporters including Renal OCT2, is crucial for drug metabolism. However, none of the identified compounds have been found to be OCT2-substrates [39]. In order to determine the total clearance of the compounds, both hepatic and renal clearance were measured [40], and the results are presented in Table 4. Overall, the table provides important information on the ADME properties of the identified compounds in CSEE extract, which can help in predicting their pharmacological activity and potential use in drug development.

**Figure 2:**
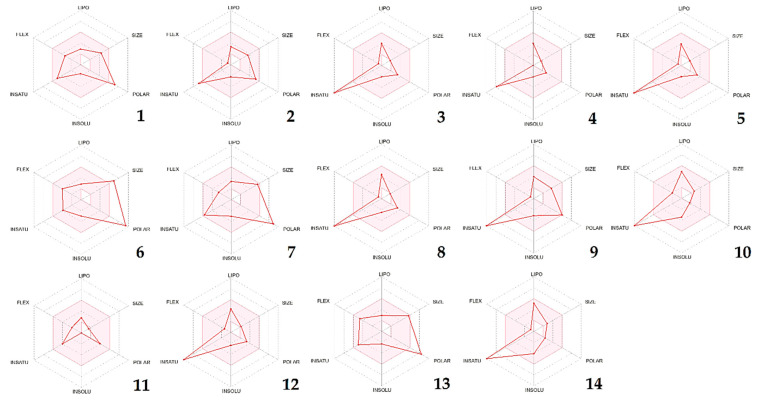
CSEE compounds’ bioavailability radars based on six physicochemical properties (lipophilicity, size, polarity, solubility, flexibility, and saturation). Note: **(1)** Chlorogenic acid, **(2)** Catechin, **(3)** 4-Hydroxybenzoic acid, **(4)** Orcinol, **(5)** 3-Hydroxybenzoic acid, **(6)** Naringin, **(7)** Salicylic acid, **(8)** Quercetin 3-*O*-β-D-glucoside, **(9)** Quercetin, **(10)** Chalcone, **(11)** Succinic acid, **(12)** 2-hydroxycinnamic acid, **(13)** Phloridzin dihydrate, **(14)** Flavone.

To ascertain the plausibility of oral bioavailability of the recognized phytoconstituents, six physicochemical attributes were taken into account and represented via bioavailability radars. These properties included lipophilicity, size, polarity, solubility, flexibility, and saturation, each of which plays a crucial role in determining the ability of a molecule to be absorbed and utilized within the body. Bioavailability radars of the identified compounds are presented in Figure 2, which provides an important visualization of their potential for oral bioavailability. The pink zone depicted on the radar denotes the region in which the molecule’s graphical representation must fit entirely to be classified as drug-like. This is an important aspect to consider when evaluating the potential of a molecule to be used as a therapeutic drug, as it indicates the likelihood of the molecule being absorbed and distributed effectively within the body. In this case, all the physicochemical descriptors of the given phytoconstituents lie within the pink radar region of the bioavailability radar, except for unsaturation in molecules 2, 3, 4, 5, 8, 9, 10, 12, and 14, which indicates that they may have too many double bonds or unsaturated groups in their chemical structures. Additionally, compounds 1, 6, 7, and 13 do not adhere to the polarity rule, implying that these compounds may be too polar to cross the lipid membranes in the gut and may thus have reduced bioavailability.

**Figure 3:**
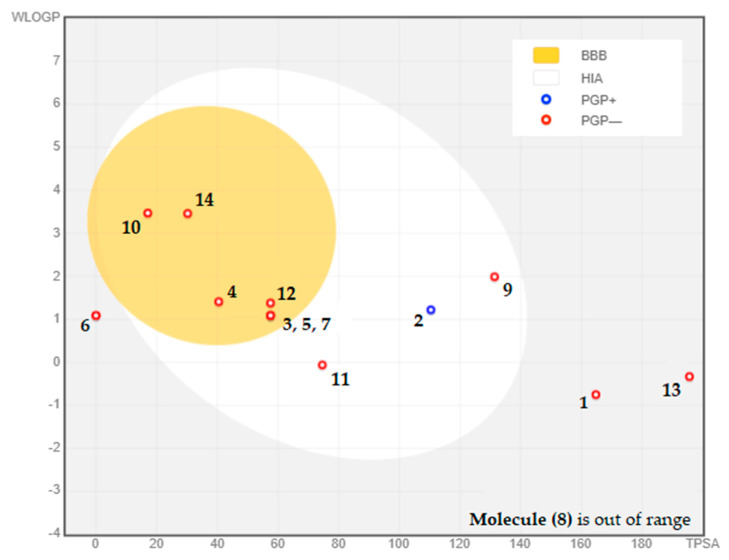
BOILED-EGG model used to assess the composition of CSEE in terms of blood-brain barrier permeability, gastrointestinal absorption, and whether the molecules act as substrates or inhibitors of P-glycoprotein. **(1)** Chlorogenic acid, **(2)** Catechin, **(3)** 4-Hydroxybenzoic acid, **(4)** Orcinol, **(5)** 3-Hydroxybenzoic acid, **(6)** Naringin, **(7)** Salicylic acid, **(8)** Quercetin 3-*O*-β-D-glucoside, **(9)** Quercetin, **(10)** Chalcone, **(11)** Succinic acid, **(12)** 2-hydroxycinnamic acid, **(13)** Phloridzin dihydrate, **(14)** Flavone.

The BOILED-Egg model is a tool that allows for a preliminary assessment of a molecule’s ability to be absorbed by the intestines and cross the blood-brain barrier. This is determined based on two key factors: lipophilicity, as measured by WLOGP, and polarity, as measured by TPSA [41]. The resulting model is depicted visually, with the white area representing molecules that are more likely to be absorbed by the intestines, while the yellow area within the yolk represents molecules that are more likely to cross the blood-brain barrier [41]. The dots in the diagram are color-coded to indicate whether a molecule is a substrate or non-substrate for P-glycoprotein. Substrates are shown in blue, while non-substrates are shown in red. In this case, seven phytocompounds (4-Hydroxybenzoic acid, Orcinol, 3-Hydroxybenzoic acid, Salicylic acid, Chalcone, 2-hydroxycinnamic acid, and Flavone) have been identified as having high levels of absorption and being able to cross the blood-brain barrier effectively. Moreover, the analysis revealed that all these compounds are non-substrates for P-glycoprotein, with Catechin (2) being the exception in Figure 3.

### 2.4. PASS Prediction

Table 5 provides the PASS prediction (Prediction of Activity Spectra for Substances) of major compounds found in CSEE, along with their biological activities such as antioxidant. Table 5 presents the PASS prediction of the primary compounds present in CSEE and their various biological activities, including antioxidant, antibacterial, antifungal, and antineoplastic (specifically breast cancer). PASS is a computer program that predicts the biological activity spectra of organic compounds based on their structural formulae. The table shows the probability of each compound being active or inactive in each biological activity category, with Pa indicating the probability of being active and Pi indicating the probability of being inactive. A value close to 1 indicates high probability of activity, whereas a value close to 0 indicates low probability of activity. The results show that some compounds have high probability of activity in multiple categories. For example, quercetin 3-*O*-β-D-glucoside has high probability of activity in antioxidant, antibacterial, antifungal, and antineoplastic (breast cancer) categories. Naringin also has high probability of activity in antioxidant, antibacterial, and antifungal categories.

### 2.5. In Silico Toxicity Prediction (Using Pro-Tox II)

The objective of the current study was to examine the potential toxicity of 14 compounds found in CSEE using computational models. The LD50 values and predicted toxicity endpoints were determined for each compound, and the hazard classes according to GHS classification were also provided (Table 6) [42]. The LD50 values indicated that compounds **1**, **2**, **3**, **5**, **8**, **10**, **11**, **12**, **13**, and **14** have low acute toxicity, while compounds **4** and **9** have higher acute toxicity. The GHS hazard classes ranged from III to VI, with most of the compounds falling into hazard classes IV and V. These results suggest that while the majority of the compounds in CSEE are not highly toxic, some may pose a potential hazard to human health. The predicted toxicity endpoints of the compounds revealed that hepatotoxicity, mutagenicity, carcinogenicity, immunotoxicity, and cytotoxicity are possible health effects associated with exposure to CSEE compounds. The highest probabilities of hepatotoxicity and mutagenicity were observed for compounds **4** and **6**. Compound **6** was also predicted to have a high probability of being carcinogenic, while compounds **4** and **14** were predicted to have moderate probabilities of being carcinogenic. Immunotoxicity was predicted for compounds **4**, **6**, and **7**, with a high probability for compound **6**. Cytotoxicity was predicted for most of the compounds, with the highest probabilities observed for compounds **1**, **5**, **6**, and **7**. It should be emphasized that the outcomes obtained in this investigation are reliant on computational models, and additional empirical research is necessary to validate the real toxicity of these constituents in humans. Nevertheless, the findings of this study offer valuable insights into the plausible health implications related to the exposure to CSEE components. The identification of the specific compounds responsible for these toxic effects can aid in the development of strategies to minimize the potential health risks associated with the consumption of CSEE.

### 2.6. Experimental Validation of the Tested Biological Activities

- Antioxidant activity

Several methods were employed to evaluate the antioxidant potential of the compounds, which are primarily based on their ability to reduce and trap free radicals, serving as a measure of their antioxidant capacity. However, the mechanism of action of each antioxidant activity is classified quantitatively, depending on how the applied compounds halt chain-breaking reactions. The results of these evaluations were presented in Table 7. According to the DPPH test, the CSEE extract was able to reduce the stable violet DPPH radical to yellow DPPH-H, with an IC_IC50_ of 302.78 ± 7.55 µg/mL, indicating strong antioxidant activity. Despite this, when compared to the pure reference antioxidant ascorbic acid (260.24 ± 6.45 µg/mL), the tested samples showed even higher levels of antioxidant activity. The effectiveness of the CSEE extract in inhibiting lipid peroxidation activity was evaluated using the β-carotene bleaching test. The results indicated that the CSEE extract demonstrated strong antioxidant activity with an IC_50_ value of 352.06 ± 12.16 µg/mL. Furthermore, it was observed that the CSEE extract exhibited a greater antioxidant activity compared to the standard Butylated hydroxytoluene (BHT), as indicated by a value of 29.23 ± 9.34 µg/mL.

In addition, ABTS assay was performed, where the ability of the extract to scavenging the ABTS•+. The CSEE extract exhibited a strong scavenging activity against ABTS radicals with an IC_50_ value of 48.13 ± 3.66 µg/mL. However, this value was more important when compared to the pure reference antioxidant ascorbic acid (8.23 ± 0.97 µg/mL). In addition, results showed that the CSEE exhibited the highest TAC with 165 ± 7.66 µg/mL. These findings were in accordance with previous studies [43,44,45].

In general, the antioxidant activity of plant extracts is influenced by the interaction of all the chemical components present, which can either act together or in opposition to one another. Several studies have shown a correlation between the antioxidant capacity of plants and their content of polyphenols and flavonoids [46,47]. The current study found that the high antioxidant activity of *C. siliqua* was due to its abundance of polyphenols and flavonoids. There was a positive correlation between the phenolic and flavonoid content and the DPPH activity. Flavonoids with certain structures, in particular, can act as donors of protons or electrons, which explains their positive correlation with the antioxidant activity [35,48].

b. Antibacterial and Antifungal Properties of CSEE

The well diffusion technique was implemented to measure the inhibition zone diameters, whereas the microdilution method was employed to determine the MIC, MBC, and MFC of CSEE (Table 8 and Table 9). It has been suggested that plant extracts are considered active when their inhibition zone diameter is ≥10 mm [49]. The plant extract was found to have antimicrobial activity against all tested bacterial and fungal strains, with inhibition zones diameters ranging from 18 to 28 mm. The largest zone of inhibition (IZ = 28 mm) was noted in the case of *E. coli*, whereas the smallest zone of inhibition (IZ = 18 mm) was observed in the case of *P. aeruginosa*. According to the data, the microdilution results showed that CSEE exhibited an inhibitory effect against all the tested bacteria and moderated effect against fungal strains with MIC values of 0.35 μL/mL and 10 μL/mL for all tested bacterial and fungal strains, respectively. Moreover, the results showed that the studied extract showed bactericidal and fungicidal potentials, with MBC values ranging from 0.35 to 0.70 μL/mL and MFC values of 10 μL/mL. These results are in accordance with several investigations which reported that *C. siliqua* extracts have a strong antimicrobial potential against a large range of microbes, including multidrug-resistant bacteria and fungal strains [50,51,52].

These results can be linked to the chemical composition of the CSEE that showed a high content of polyphenols. Indeed, the antimicrobial potential of plant polyphenols has been widely investigated against human pathogens in order to develop new antimicrobials [53]. Different mechanisms are suggested to explain the antimicrobial potential of polyphenols. Several studies reported that highly oxidized phenolic compounds exert a greater inhibitory effect on microorganisms. Moreover, it has been reported that phenolics may induce a loss of protein functions by serving as a source of stable free radicals that bind with proteins in an irreversible way. Polyphenols can act via other mechanisms, such as binding to adhesins located on the surface of the microbial cell, complexing with metal ions, or interacting with some substrates, rendering them inaccessible for microorganisms [54,55]. Our findings showed that CSEE contains 29.61 ± 0.36 g CE/100 DW of condensed tannins. Several studies reported that tannins affect the membrane permeability, and an increase of tannins concentration is accompanied with a decrease in the permeability of the membrane [56]. In this respect, a recent study demonstrated that tannins engendered an aggregation and precipitation of *S. aureus* cells, which consequently induced a decrease in membrane permeability and a reduction of oxygen mass transfer into cells [57]. Furthermore, the results of the HPLC-DAD profile of CSEE showed that the majority of the compounds of the extract belong mostly to flavonoids (Table 1). Indeed, flavonoids are known to possess strong antimicrobial potential by exhibiting protection against plant pathogens and, as a result, they can exhibit efficacy in the mitigation of human pathogens as well [58]. Flavonoids exert their antimicrobial effect through various mechanisms including the inhibition of cell envelop synthesis, the inhibition of efflux pump, the inhibition of nucleic acid synthesis, the inhibition of virulence enzymes, the inhibition of biofilm formation, as well as membrane disruption [59].

c. Cytotoxicity of CSEE Against Breast Cancer Cell Lines (MCF-7, MDA-MB-231, and MDA-MB-436)

**Figure 4:**
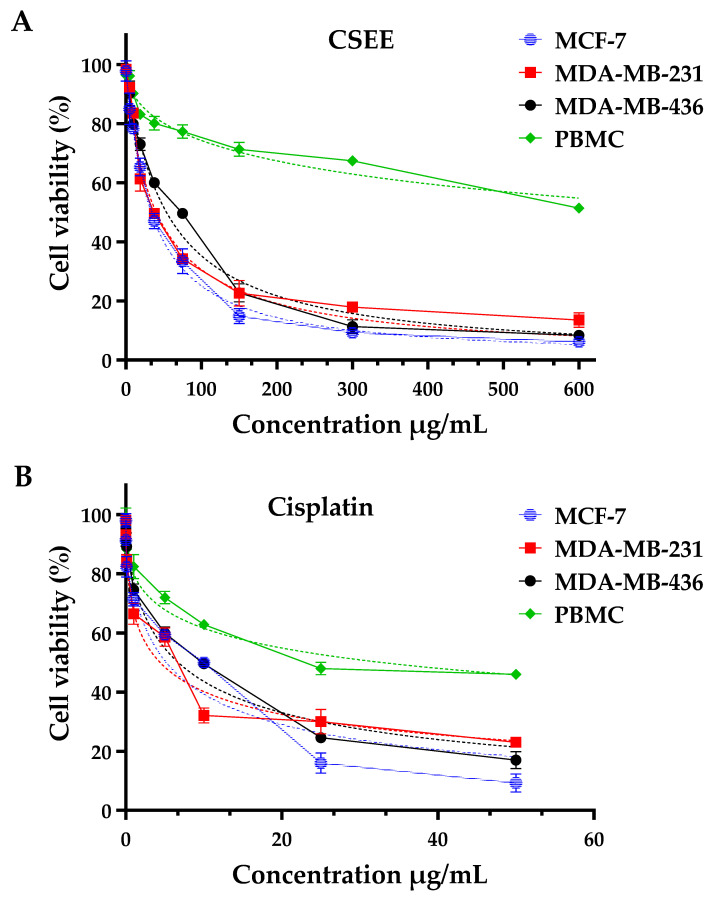
Using the MTT test, evaluation of cell viability in MCF-7, MDA-MB-231, MDA-MB-436, and PBMC cells treated with *C. siliqua* ethanolic extract (**A**) and cisplatin (positive control, (**B**)) for 72 h.

*C. siliqua* is a plant widely used in folk medicine for its bioactive properties, especially its polyphenols, which have been identified as having beneficial potential against human diseases such as cancer and metastasis [60,61]. Different extracts of *C. siliqua* have been tested in vitro against several tumor cells [21,62,63,64]. In our study, we investigated the cytotoxic power of CSEE against breast cancer (MCF-7) and the two metastatic adenocarcinoma lines (MDA-MB-231, and MDA-MB-436) using the MTT assay (Figure 4). Cisplatin was employed as a positive control. Table 10 displays that the CSEE had varying levels of cytotoxicity against different cell lines. MCF-7 was found to be the most sensitive cell line with an IC_50_ value of 32.44 ± 5.23 µg/mL, whereas MDA-MB-231 and MDA-MB-436 were less sensitive with IC_50_ values of 40.05 ± 3.21 µg/mL and 53.55 ± 5.35 µg/mL, respectively. Our findings were superior to those of Custódio et al. (2011) [62], who observed the effect of *C. siliqua* extract on MDA-MB-231, and after 24 h of incubation a moderate efficacy was reported with an IC_50_ level greater than 400 µg/mL.

The requirement to exhibit a minimal amount of cytotoxicity is due to the fact that PBMCs are the first normal cell populations to come into contact with anticancer medicines employed in the conventional intravenous chemotherapy of patients. In fact, we used the MTT test to assess how CSEE affected the vitality of PBMCs. With an IC50 > 890 µg/mL, the results obtained indicated little cytotoxicity against PBMCs. As a matter of fact, in contrast to cisplatin, CSEE has a greater cytotoxic effect on tumor cells than on PBMCs. These findings point to these compounds’ highly selective ability to destroy tumor cell lines while having no negative effects on normal cells. The results indicate that CSEE has a higher IC50 value (i.e., lower potency) than cisplatin in all three cancer cell lines, indicating that it is less effective in inhibiting cancer cell growth. However, the SI values of CSEE are higher than those of cisplatin, indicating that it is more selective in inhibiting cancer cells while having less effect on PBMC. This is a positive and very promising finding, as it suggests that CSEE may have a more targeted and less toxic effect on cancer cells compared to cisplatin, which is known to have significant side effects.

d. Genotoxicity Evaluation of CSEE on Rat Leukocytes

The initial test performed to evaluate the health safety of a substance, medicine, or nutraceutical is commonly believed to be the genotoxicity test [65]. One highly accurate and rapid microscopic technique for assessing DNA damage—including single and double strand breaks, oxidative damage, and DNA-protein interactions, in both prokaryotic and eukaryotic cells in vitro and in vivo—is the comet assay, also known as Single Cell Gel Electrophoresis (SCGE), which employs agarose microgel electrophoresis [66,67,68,69]. The alkaline form of the comet assay was developed especially for detecting single-strand breaks and alkali-labile sites [70].

**Figure 5:**
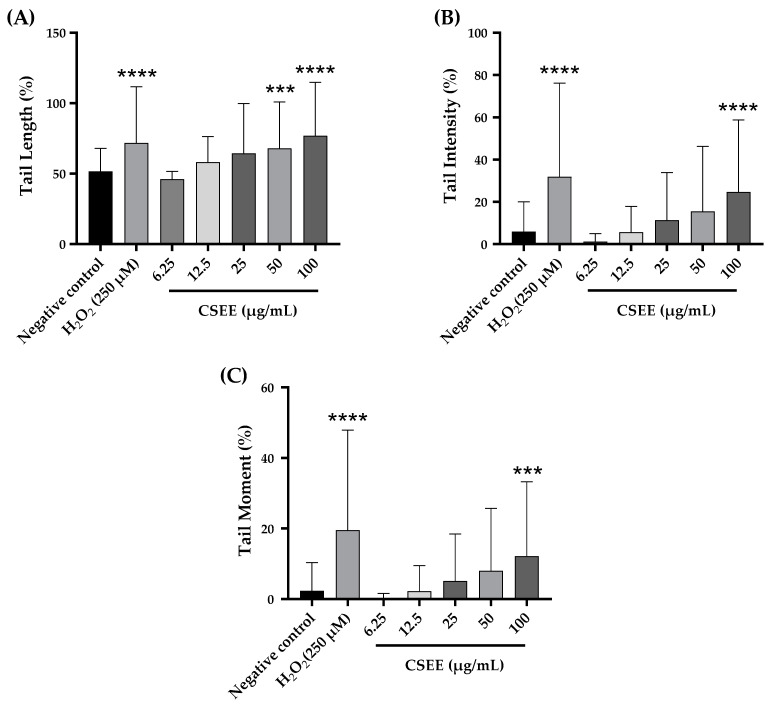
Assessment of the impact of varied concentrations of *C. siliqua* ethanolic extract (CSEE) on (**A**) DNA tail length, (**B**) the percentage of tail intensity, and (**C**) DNA tail moment in rat leukocytes. Results presented as mean ± SEM (50 cells × 2). **** *p* < 0.0001 compared to the negative control group, *** *p* < 0.001 compared to the negative control group.

In terms of the impact on the proportion of DNA in the tail and the tail moment (as illustrated in Figure 5), it was observed that the concentrations of 6.25, 12.5, 25, and 50 µg/mL of *C. siliqua* ethanolic extract did not result in any DNA harm according to our findings. However, the 100 µg/mL concentration showed a significant genotoxic effect compared to the negative control. In summary, we can conclude that the CSEE was genotoxic over a dose of 100 μg/mL. The extracts’ mode of action is currently unknown. Numerous investigations have demonstrated that plant extracts can have pro- or anti-mutagenic and antioxidant or pro-oxidant effects, mostly dependent on the dose utilized [71,72]. Flavonoids have been shown to have multiple biological actions, demonstrating that they have the potential to be both mutagenic and protective at high doses [72,73,74]. The extract’s pro-mutagenic action, which is reflected in its pro-oxidant activity in producing free radicals that damage DNA, may be influenced by high quantities of flavonoids in elevated concentrations of the extract. In addition, when flavonoids are present at higher concentrations, they may be causing damage to the genetic material by intercalating into DNA, inhibiting enzymes that are associated with DNA such as topoisomerase II, blocking important enzymes that are involved in hormone metabolism, and changing the behavior of other significant enzymes, which can lead to the production of clastogenic effects [75,76]. Several polyphenols and flavonoids, including vanillic acid, caffeic acid, chlorogenic acid, ferulic acid, and quercetin are well-known antioxidants; yet, depending on the dose, they can also be pro-oxidants, which is why they are a substantial contributor to DNA damage [77,78,79,80,81].

## 3. Materials and Methods

### 3.1. Plant Origin, and Extraction Procedure

The leaves used in this research were sourced from a recently discovered carob tree variety that is indigenous to Eastern Morocco. This unique type of carob was identified and registered by Prof. Dr. Abdelbasset Berrichi, who is affiliated with the Faculty of Sciences, Mohammed Premier University, located in Oujda, Morocco. The specific variety used in the research is known as “Rahma”, and it is a hermaphrodite plant species. It is also considered as a conservation variety, indicating that it is a primitive breed that has adapted naturally to the local and regional environmental conditions. However, these types of agricultural varieties are currently facing the threat of genetic erosion, emphasizing the importance of their valorization and preservation.

Leaves were harvested from *C. siliqua* L. var. Rahma trees growing in the nursery of the Faculty of Sciences, Mohammed Premier University, located in Oujda, Morocco. The Department of Biology at the same university identified and assigned a voucher specimen code ***** to the leaves. First, the leaves were processed in a commercial blender, and a quantity of 10 g of the resulting powder was combined with 50 mL of 99% ethanol. The mixture was filtered with a vacuum pump, and the solvent was removed through evaporation in a rotary evaporator under specific conditions of 250 bar pressure, 60 °C temperature, and 150 rpm. The outcome was the extract CSEE, which was then kept in storage at a temperature of −4 °C until it was needed.

### 3.2. Analysis of Phenolic Compounds (HPLC-DAD)

Advanced analytical methods were utilized to examine the ethanolic extract. These included a HPLC/DAD system manufactured by Waters Corporation, Milford, MA, USA, which utilizes high-performance liquid chromatography coupled with a diode array detector. The information generated from this analysis was then managed and analyzed using the empower software [82]. The prepared samples (20 µL) were injected in a Zorbax XDB-C18 (5 µm porosity, 250 × 4.6 mm) using an automatic injection system with an elution gradient of 0–25 min at 20% B, 25–30 min at 100% B, and 30–35 min at 20% B. The signals obtained were integrated using an Agilent ChemStation HPLC system at a flow rate of 1 mL/min. The mobile phases used for sample elution were A (water/0.5% phosphoric acid) and B (methanol), and the separation was carried out at a constant temperature of 40 °C. Spectrophotometric measurements were taken at 280 nm. To identify the compounds present in the ethanolic extract, we compared the observed peaks’ retention time and UV spectra with those of an authentic reference solution (5 mg/mL) performed on the same column under the same conditions [82].

### 3.3. TPC, TFC, and TCT Contents

The measurement of the complete amount of polyphenols present in the *C. siliqua* extract was carried out using the Folin-Ciocalteu technique as per the reference protocol [64,83]. Initially, a solution of the extract was prepared by mixing 100 µL of the extract (2 mg/mL) with 200 µL of Folin-Ciocalteu reagent in 2 mL of distilled water. Subsequently, 1 mL of 15% sodium carbonate solution was added to the mixture and the resulting solution was kept in the dark and incubated at ambient temperature for a period of 2 h. The absorbance was determined at 765 nm utilizing a spectrophotometer. A calibration curve was constructed using gallic acid over a concentration range of 0–0.1 mg/mL. The quantity of total phenolic content was reported as milligrams of gallic acid equivalents per gram of dry extract (mg GAE/100g DW). To determine the total flavones and flavanols content, the CSEE was subjected to the colorimetric assay using aluminum chloride (AlCl_3_) following the protocol described by Frond et al. [84]. Briefly, 500 µL of each extract (2 mg/mL) was mixed with 1.5 mL of MeOH. Then, 100 µL of AlCl_3_ (10%) and 100 µL of potassium acetate (1 M) were added, and the volume was made up to 2.8 mL with distilled water. After incubation at room temperature in the dark for 30 min, the absorbance was measured at 415 nm against a blank. The vanillin method, as described by Mohti et al. (2020) [85], was utilized to determine the condensed tannin content of the CSEE. In this method, 50 µL of the extract solution was combined with 1.5 mL of vanillin (MeOH, 4%) by gentle agitation or vortexing to ensure a uniform mixture, and then 750 µL of concentrated acid (HCl) was added. The mixture obtained was subjected to incubation at ambient temperature under dark conditions for 20 min. Subsequently, the absorbance of the mixture was measured at 500 nm. The content of condensed tannins was reported as mg catechin equivalents per gram of dry extract (mg CE/100g DW), and a calibration curve was established using catechin at a level between 0 to 5 mg/mL. To ensure accurate and reproducible results, all measurements were performed in triplicate.

### 3.4. PASS, ADME, and the Prediction of the Toxicity Analysis (Pro-Tox II)

In this particular investigation, the Pharmacological Assessment of Structure Similarity (PASS) method was utilized to assess the potential pharmacological activity of the primary chemical constituents present in the extract of CSEE [86]. The molecules were first transformed into SMILES format using ChemDraw, and then examined using the PASS online application to predict their probable activity (Pa) and likely inactivity (Pi) [87,88]. Moreover, SwissADME (http://www.swissadme.ch/ accessed on 19 April 2023) and pkCSM (http://biosig.unimelb.edu.au/pkcsm/ accessed on 19 April 2023) webservers were utilized to assess the physicochemical properties, drug similarity, and pharmacokinetic properties of the compounds [40,89,90]. To evaluate toxicity levels, the Protox II online tool (https://tox-new.charite.de/protox II/, accessed on 19 April 2023) was utilized to provide data on LD_50_ values, toxicity class, and various toxicological endpoints [42]. The utilization of these methodologies and instrumentation yielded significant revelations regarding the potential therapeutic applications and adverse effects associated with the principal chemical compounds identified within CSEE.

### 3.5. Antioxidant Activity

#### 3.5.1. 2,2-Diphenyl-1-Picrylhydrazil Free Radical Scavenging Assay

The antioxidant capacity of CSEE was determined through a modified DPPH method based on previously established procedures [91,92]. To prepare the DPPH-MeOH solution, 2 mg of DPPH was dissolved in 100 mL of methanol. A set of CSEE solutions were prepared at various concentrations ranging from 5 to 500 µg/mL. Afterwards, 2.5 mL of the DPPH mixture was added to each of the different CSEE solutions, and the total volume was adjusted to 3 mL. After the mixture was incubated at room temperature for 30 min, its absorbance was measured at 517 nm compared to a blank. The formula employed to determine the percentage of DPPH free radical scavenging activity was as follows:

$$Free\;Radical\;Scavenging\left(\%\right)=\left[\left(\frac{A_{blank}-A_{sample}}{A_{blank}}\right)\right]\times 100$$

The absorbance of the control reaction (which contained all reagents except the extract) was denoted as Ablank, while the absorbance of the extract at varying concentrations was denoted as Asample. To determine the IC_50_, the inhibition percentage was plotted against the extract concentrations on a graph. As a positive control, ascorbic acid was used.

#### 3.5.2. β-Carotene Bleaching Assay

In order to evaluate the antioxidant activity of CSEE, the β-Carotene assay based on bleaching was employed, following a modified version of the method described in previous studies [93,94]. Initially, a mixture of 2 mg of β-carotene in 10 mL of chloroform was prepared and mixed with a solution of 20 mg of linoleic acid and 200 mg of Tween-80. The chloroform was removed using a rotavapor at 40 °C, and 100 mL of distilled water was added with vigorous shaking. The derived samples were distributed in triplicate fashion across a 96-well plate and stored in a light-restricted environment at 25 °C for a period of 30 min. Upon the addition of CSEE solution (t0), the samples were promptly analyzed via spectrophotometry at a wavelength of 470 nm. A second reading was taken following a two-hour incubation (t1), with both measurements compared against a blank reading that incorporated all components of the CSEE solution except for β-carotene. BHA served as the standard reference in this experiment. To obtain precise and dependable outcomes, the residual color (%) was ascertained utilizing the following formula:

$$Residual\;color\left(\%\right)=\left[\left(\frac{Initial\;OD-Sample\;OD}{Initial\;OD}\right)\right]\times 100$$

#### 3.5.3. ABTS Scavenging Activity Assay

The ABTS radical scavenging ability of CSEE was evaluated using a modified version of the method described by Nakyai et al. (2021) [91]. To generate ABTS•+ radical cation, the ABTS solution was combined with 2.45 mM potassium persulfate and kept in the dark at ambient temperature for 16–18 h. The resultant solution was diluted with ethanol to attain an absorbance of 0.70 ± 0.02 at 750 nm. The L-ascorbic acid was utilized as a positive control. To perform the ABTS assay, 20 µL of the test sample was combined with 200 µL of the ABTS•+ solution that had been previously diluted. The resulting mixture was then left in the dark at room temperature for 10 min, after which the absorbance was measured at 734 nm using a microplate reader. The percentage of ABTS radical cation scavenging activity was determined using a method similar to that used for the DPPH assay.

#### 3.5.4. Total Antioxidant Capacity

The determination of antioxidant activity in the sample was conducted utilizing the phosphor-molybdenum methodology expounded in reference [92]. In accordance with this technique, the sample extract/standard solution was mixed with a reagent solution comprising 0.6 M sulfuric acid, 28 mM sodium phosphate, and 4 mM ammonium molybdate, followed by incubation at 95 °C for a duration of 90 min and cooling to room temperature. The resultant solution’s absorbance was gauged at 695 nm, and the outcome was communicated as ascorbic acid equivalents utilizing a standard curve that was established with ascorbic acid [95]. The blank solution, which excluded the test sample, encompassed all reagents, while the experiments were performed thrice to validate the precision and repeatability of the outcomes.

### 3.6. Antibacterial Activity

#### 3.6.1. Bacterial Strains and Growth Conditions

In this study, the antibacterial properties of CSEE extract were assessed against five distinct bacterial strains obtained from the Laboratory of Microbial Biotechnology at the Faculty of Science in Oujda, Morocco. These bacterial strains encompassed two types of Gram-positive bacteria, specifically *Staphylococcus aureus* (ATCC 6538) and *Enterococcus faecalis* (ATCC 29212), and three types of Gram-negative bacteria, namely *Escherichia coli* (ATCC 10536), *Escherichia vekanda*, and *Pseudomonas aeruginosa* (ATCC 15442). The bacterial strains were cultured on Luria-Bertani-Agar (LBA) medium and were subsequently incubated at 37 °C for a duration of 24 h. Prior to the administration of the CSEE extract, the bacterial concentration was quantified and regulated to 106 cells/mL using a UV-Visible spectrophotometer at 620 nm.

#### 3.6.2. Disc Diffusion Method

To determine the antimicrobial activity of CSEE against mycobacteria, the disc diffusion method was used following the National Committee for Clinical Laboratory Standards (NCCLS) guidelines [96]. This method is effective in measuring the substance’s ability to inhibit mycobacterial growth [97]. A microbial suspension containing 10^8^ germs/mL in physiological saline was inoculated on Mueller-Hinton agar plates [98]. A blank paper disk containing 10 µL of CSEE was then placed on the cultured media’s surface. The plates were then incubated at 37 °C for 24 h, and inhibition diameters were measured in millimeters using a ruler. The experiment was conducted in triplicate, with Imipeneme (10 µg/disc) or Amoxicillin (25 µg/disc) disks used as the positive control.

#### 3.6.3. Determination of the MIC, and the MBC

The assessment of the effectiveness of substances that fight against microbes requires the determination of the minimum inhibitory concentration (MIC). The study discussed here used the resazurin micro-titer assay to measure the MIC of an extract from *C. siliqua*. This assay involves the use of a color-changing substance called resazurin, which is reduced by active cells, causing a color shift from blue to pink. The antimicrobial agent was added to each well of a 96-well microplate at different concentrations, and a standardized inoculum of the bacteria being tested was added to each well. The microplates were then incubated for 24 h at 37 °C, followed by the addition of resazurin to each well, as delineated in reference [99]. The plates were further incubated for 4–6 h until a color change was observed. The MIC was determined as the lowest concentration of the antimicrobial agent that resulted in no color change, indicating the absence of viable bacteria. Controls were used to ensure the accuracy of the results. The minimum bactericidal concentration (MBC) was determined by taking a sample from the negative wells and plating it onto Mueller Hinton Agar medium plates. The plates were then incubated at 37 °C for 24 h, and the lowest concentration of the extract that did not result in bacterial growth was determined to be the MBC, as depicted in reference [99]. The experiment was repeated three times to ensure reproducibility. This section does not discuss antifungal activity.

### 3.7. Antifungal Activity

#### 3.7.1. Selection and Source of Bacterial Strains

To determine the antifungal activity of the CSEE under investigation, two pure strains of fungi, namely *Geotrichum candidum* and *Candida albicans* were utilized. They were sourced from the same previously mentioned laboratory.

#### 3.7.2. Inoculum Preparation and Disk Diffusion Technique

The fungal species *G. candidum* was cultured on PDA medium from BIOKAR at 25 °C for a week, and the spore concentration was adjusted to 2 × 10^6^ spores/mL using a Thoma cell hemacytometer. Similarly, *C. albicans* was cultured on YPD medium at 25 °C for 48 h, and the cell concentration was adjusted to 10^6^ cells/mL for each yeast strain. To evaluate the antimicrobial activity of CSEE against mycobacteria, the disc diffusion method recommended by NCCLS was employed, as outlined in Section 3.6.2. [97].

#### 3.7.3. Determination of the MIC, and the MFC

The experiment was conducted in 96-well microplates, with each well containing a different concentration (0.18 to 89.6 μL/mL) of the antifungal agent. There were three replicates for each concentration, and a standardized amount of the fungal strain was incorporated to each well. After 48 h of incubation at 25 °C, resazurin was added to the microplate wells, and the plates were further incubated for 2 h until the blue color of the solution turned pink. The MIC was defined as the lowest concentration of the antifungal agent that did not cause a color change, indicating that the fungi were not viable. The accuracy of the results was confirmed by using positive and negative controls. To determine the MFC, samples from the wells with no visible growth after the MIC test were inoculated onto YEG and PDA medium plates and incubated for 48 to 72 h. The MFC was determined as the extract concentration that completely prevented the visible growth of fungi, demonstrating that the extract has fungicidal properties rather than simply inhibiting fungal growth.

### 3.8. Cytotoxicity against Breast Cancer Cell Lines

#### 3.8.1. Cell Culture

Two types of breast cancer cells, including MCF-7 cells that are estrogen receptor-positive and MDA-MB-231 and MDA-MB-436 cells that are estrogen receptor-negative, were utilized in the study. The cells were cultured in Dulbecco’s Minimum Essential Medium (DMEM) supplemented with 10% fetal bovine serum (FBS) and 50 µg/mL gentamicin at 37 °C with 5% CO_2_ in a humidified atmosphere to ensure their survival. The cells were subcultured in 25 cm^2^ tissue culture flasks for continuous growth. The study used cells in the exponential growth phase to conduct the cell viability analysis.

#### 3.8.2. Cell Viability by MTT Assay

To determine whether CSEE could inhibit the proliferation of cancer cells, we used the MTT assay, following the method described in references [92,100]. Exponentially growing MCF-7, MDA-MB-231, and MDA-MB-436 cells were seeded onto 96-well plates at a density of 10^4^ cells per well in 100 µL of medium and allowed to adhere for 24 h. Various concentrations of CSEE were obtained by solubilizing it in 0.1% DMSO and serially diluting it with medium. Different concentrations of CSEE were prepared by dissolving it in 0.1% DMSO and diluting it with medium. The cells were then exposed to varying concentrations of CSEE for 72 h. The control group cells were only given medium containing 0.1% DMSO. After replacing the medium with 200 µL of culture medium, 20 µL of MTT reagent (5 mg/mL MTT in PBS) was added and incubated for 4 h at 37 °C. After removing the medium, 100 µL of DMSO was added, and the absorbance was measured at 540 nm using a microplate reader (Synergy HT Multi-Detection microplate reader, Bio-Tek, Winooski, VT, USA) to calculate the percentage of cell viability [101]. The study assessed the impact of CSEE on cell viability by measuring absorbance using the following equation.

$$Cell\;viability\left(\%\right)=100-\left[\left(\frac{A_{0}-A_{t}}{A_{0}}\right)\times 100\right])$$

A_0_ = Absorbance of cells treated with 0.1% DMSO medium, and A_t_ = Absorbance of cells treated with CSEE at various concentrations. A negative control group was given 0.1% DMSO in the medium, and GraphPad Prism 8.01 software was used to calculate IC50 values, with cisplatin as the standard. Ethical approval was granted by the Research Ethics Committee (03/22-LAPABE-10 and 4 March 2022) prior to the experiment. To assess the cytotoxic effects of CSEE on peripheral blood mononuclear cells (PBMCs), the same conditions and concentrations used for tumor cells were utilized. PBMCs were isolated from human blood samples using Ficollhypaque density centrifugation as per the manufacturer’s instructions (Capricorn Scientific, Ebsdorfergrund, Germany).

### 3.9. Genotoxic Effect

#### 3.9.1. Blood Sample Collection and Treatment of Cells

Pentobarbital anesthesia was utilized to anesthetize the rats prior to the collection of retro-orbital vein blood samples using tubes containing heparin. Fresh blood was collected from a male Wistar rat and diluted with 2 mL of PBS without Ca^2+^ and Mg^2+^ (137 mM NaCl; 2.7 mM KCl; 10 mM Na_2_HPO_4_; 1.76 mM KH_2_PO_4_; pH 7.4). The samples were then exposed to the blood cells, with different concentrations (100, 50, 25, 12.5, and 6.25 µg/mL) achieved by dissolving them in PBS. After exposure for 1 h at 37 °C, 10 µL of blood cells were analyzed. The negative control was exposed to PBS, while hydrogen peroxide (250 µmol/L) was used as a positive control.

#### 3.9.2. Comet Assay

The protocol for the alkaline comet test, as outlined by Ouahhoud et al. (2022) [102], underwent minor modifications before it was conducted. After treatment, the suspension was centrifuged at 4500 rpm for 10 min, and the leukocyte-containing pellet was dissolved in 1 mL of PBS following the removal of the supernatant. The washing process was repeated three times, and the resulting pellet was dissolved in LMP agarose (0.5% *w*/*v* in PBS) before being applied to a slide coated with NMP agarose (1.5% *w*/*v*). The slides were exposed to a lysis solution (2.5 M NaCl, 100 mM Na_2_-EDTA, 20 mM Tris, 300 mM NaOH, 1% N-lauroylsarcosine sodium, 10% DMSO, and 1% Triton X-100) for 5 min, and then incubated for 1 h in the dark at 4 °C. After washing with double distilled water, the slides were subjected to horizontal gel electrophoresis using an electrophoresis solution (300 mM NaOH and 1 mM Na_2_EDTA, pH 13) for 20 min at a constant current of 300 mA and a set voltage of 25 V. Throughout the electrophoresis, the temperature of the electrophoresis solution was maintained at 4 °C. The slides were then neutralized in a Trizma buffer solution (400 mM Trizma solution adjusted to pH 7.5 by HCl) and the process was repeated three times. The comets were visualized using the ethidium bromide method as described by Singh et al. (1988) [70]. In fact, each slide was coated with 50 µL of ethidium bromide stain before being covered with a fresh cover slip. Excess stain from the slides’ edges and back should be wiped away before viewing.

#### 3.9.3. Examination under Microscope

The ethidium bromide-stained slides were examined and captured using the red channel of a fluorescence microscope called ZOE Cell Imager, which uses excitation of 556/20 nm and emission of 615/61 nm. To quantify the extent of DNA damage, an image analysis tool linked with processing software was utilized. For this study, we employed the commercial Comet Assay IV software, which allowed us to measure various parameters related to DNA damage such as the proportion of DNA in the head and the proportion of DNA in the tail, the tail moment (the product of the length of the tail and the proportion of DNA in the tail), the tail area, etc. [103]. Two sets of replicates were conducted for each sample, and 50 cells were selected per replicate. Comets must be arbitrarily chosen and ought to encompass the entire gel. Comets spotted in overlapping, air-bubbled, or edgy regions should be ignored.

## 4. Conclusions

The present study showed that CSEE has a rich and diverse range of phenolic compounds and flavonoids, such as naringin, succinic acid, 2-hydroxycinnamic acid, flavone, phloridzin dihydrate, 3-hydroxybenzoic acid, orcinol, and syringic acid. The presence of naringin in the extract was particularly noteworthy. Additionally, the results indicated that CSEE has potent antioxidant properties. To further investigate the effects of CSEE on cancer cells, three human breast cancer cell lines (MCF-7, MDA-MB-231, and MDA-MB-436) were studied. CSEE demonstrated significant cytotoxicity against the three cell lines in a dose-dependent manner, with the MCF-7 cell line being more sensitive. However, CSEE did not exhibit cytotoxicity towards normal cells (PBMCs). Nonetheless, it was found that the extract had a genotoxic potential at concentrations up to 100 µg/mL. This study concludes that CSEE can be used as a natural and healthy source of bioactive substances for preventive and therapeutic purposes without causing any toxicity.

## Figures and Tables

**Table 1:** Phenolic profile of the ethanolic extract, CSEE, using HPLC-DAD.

N°	Compounds	Formula	Group	RT (min)	Area	%Area
**1**	Gallic acid	C_7_H_6_O_5_	Phenolic acids	3.133	940.85	0.70
**2**	**Chlorogenic acid**	**C_16_H_18_O_9_**	**Hydroxycinnamic acids**	**9.636**	**4163.66**	**3.12**
**3**	**Catechin**	**C_15_H_14_O_6_**	**Flavonoids (Flavan-3-ols)**	**10.614**	**3993.53**	**2.99**
**4**	**4-Hydroxybenzoic acid**	**C_7_H_6_O_3_**	**Hydroxybenzoic acids**	**10.923**	**6061.32**	**4.54**
**5**	Catechin hydrate	C_15_H_14_O_6_•H_2_O	Flavonoids (Flavan-3-ols)	12.004	1701.96	1.27
**6**	Caffeic acid	C_9_H_8_O_4_	Hydroxycinnamic acids	12.924	926.39	0.69
**7**	Syringic acid	C_9_H_10_O_5_	Hydroxybenzoic acids	13.682	2254.99	1.69
**8**	**Orcinol**	**C_7_H_8_O_2_**	**Phenolics**	**13.920**	**7421.81**	**5.56**
**9**	Vanillic acid	C_8_H_8_O_4_	Hydroxybenzoic acids	14.91	1573.56	1.18
**10**	Vanillin	C_8_H_8_O_3_	Phenolic aldehydes	15.24	2633.05	1.97
**11**	**3-Hydroxybenzoic acid**	**C_7_H_6_O_3_**	**Hydroxybenzoic acids**	**15.94**	**6976.20**	**5.22**
**12**	**Naringin**	**C_27_H_32_O_14_**	**Flavonoids (Flavanones)**	**16.14**	**23,431.57**	**17.54**
**13**	Cinnamic acid	C_9_H_8_O_2_	Hydroxycinnamic acids	16.47	1537.51	1.15
**14**	Ferulic acid	C_10_H_10_O_4_	Hydroxycinnamic acids	16.88	2446.93	1.83
**15**	*p*-coumaric acid	C_9_H_8_O_3_	Hydroxycinnamic acids	17.69	499.77	0.37
**16**	Sinapic acid	C_11_H_12_O_5_	Hydroxycinnamic acids	18.13	1763.00	1.32
**17**	**Salicylic acid**	**C_7_H_6_O_3_**	**Hydroxybenzoic acids**	**18.42**	**6891.03**	**5.16**
**18**	Flavanone	C_15_H_12_O_2_	Flavonoids (Flavanones)	19.410	2581.39	1.93
**19**	**Quercetin 3-*O*-β-D-glucoside**	**C_21_H_20_O_12_**	**Flavonoid glycosides**	**19.763**	**3448.31**	**2.58**
**20**	Rutin	C_27_H_30_O_16_	Flavonoid glycosides	20.597	468.33	0.35
**21**	**Quercetin**	**C_15_H_10_O_7_**	**Flavonoids**	**21.283**	**4558.55**	**3.41**
**22**	**Chalcone**	**C_15_H_12_O**	**Flavonoids**	**21.730**	**4131.55**	**3.09**
**23**	**Succinic acid**	**C_4_H_6_O_4_**	**Dicarboxylic acids**	**22.023**	**16,122.19**	**12.07**
**24**	**2-hydroxycinnamic acid**	**C_9_H_8_O_4_**	**Hydroxycinnamic acids**	**22.703**	**11,096.60**	**8.31**
**25**	Rutin hydrate	C_27_H_30_O_16_•xH_2_O	Flavonoid glycosides	23.600	744.53	0.56
**26**	Kaempferol	C_15_H_10_O_6_	Flavonoids	23.988	2205.06	1.65
**27**	**Phloridzin dihydrate**	**C_21_H_28_O_12_**	**Dihydrochalcones**	**25.745**	**2819.78**	**2.11**
**28**	**Flavone**	**C_15_H_10_O_2_**	**Flavonoids (Flavones)**	**27.978**	**8316.22**	**6.23**
**29**	Apigenin	C_15_H_10_O_5_	Flavonoids (Flavones)	39.344	351.88	0.26
**30**	3-hydroxy flavone	C_15_H_10_O_3_	Flavonoids (Flavones)	41.522	1504.01	1.13

**Table 2:** Total phenolic, flavone and flavonol, and condensed tannins contents in CSEE, from Morocco. Data presented as mean ± SD, and experiments were done in triplicates (n = 3).

Extract	Total Polyphenol Content (mg GAE/100 g DW)	Total Flavone and Flavonol Content (mg RE/100 g DW)	Total Condensed Tannins (mg CE/100 DW)
C. siliqua Ethanolic Extract (CSEE)	96.98 ± 1.15	5.92 ± 0.06	29.61 ± 0.36

**DW**, dry weight; **GAE,** gallic acid equivalents; **RE**, Rutin equivalents; **CE**, catechin equivalents.

**Table 3:** Physiochemical and drug-likeness analysis of the major compounds found in CSEE. **(1)** Chlorogenic acid, **(2)** Catechin, **(3)** 4-Hydroxybenzoic acid, **(4)** Orcinol, **(5)** 3-Hydroxybenzoic acid, **(6)** Naringin, **(7)** Salicylic acid, **(8)** Quercetin 3-*O*-β-D-glucoside, **(9)** Quercetin, **(10)** Chalcone, **(11)** Succinic acid, **(12)** 2-hydroxycinnamic acid, **(13)** Phloridzin dihydrate, **(14)** Flavone.

Compound Number	HBD	HBA	TPSA (Å^2^)	Log Po/w (WLOGP)	Log S (SILICO S-IT)	Lipinski’s Rule of Five	Veber Filter
**1**	6	9	164.75	−0.75	0.40 (+++)	Yes; 1 violation: NHorOH > 5	No; 1 violation: TPSA > 140
**2**	5	6	110.38	1.22	−2.14 (+++)	Yes; 0 violation	Yes; 0 violation
**3**	2	3	57.53	1.09	−1.17 (+++)	Yes; 0 violation	Yes; 0 violation
**4**	2	2	40.46	1.41	−1.59 (+++)	Yes; 0 violation	Yes; 0 violation
**5**	2	3	57.53	1.09	−1.17 (+++)	Yes; 0 violation	Yes; 0 violation
**6**	8	14	225.06	−1.49	−0.49 (+++)	No; 3 violations: MW > 500, NorO > 10, NHorOH > 5	No; 1 violation: TPSA > 140
**7**	8	12	210.51	−0.54	−1.51 (+++)	No; 2 violations: NorO > 10, NHorOH > 5	No; 1 violation: TPSA > 140
**8**	2	3	57.53	1.09	−1.17 (+++)	Yes; 0 violation	Yes; 0 violation
**9**	5	7	131.36	1.99	−3.24 (+++)	Yes; 0 violation	Yes; 0 violation
**10**	0	1	17.07	3.47	−4.96 (++)	Yes; 0 violation	Yes; 0 violation
**11**	2	4	74.60	−0.06	0.61 (+++)	Yes; 0 violation	Yes; 0 violation
**12**	2	3	57.53	1.38	−1.28 (+++)	Yes; 0 violation	Yes; 0 violation
**13**	9	12	195.60	−0.33	−1.66 (+++)	No; 2 violations: NorO > 10, NHorOH > 5	No; 1 violation: TPSA > 140
**14**	0	2	30.21	3.46	−6.13 (+)	Yes; 0 violation	Yes; 0 violation

**HBD:** Hydrogen-Bond Donors; **HBA:** Hydrogen-Bond Acceptors; **Log Po/w:** distribution coefficient P; **Log S:** Solubility; (+++) Soluble, (++) Moderately Soluble, (+) Poorly Soluble.

**Table 4:** The (ADME) pharmacokinetic characteristics of the identified compounds present in the extract of CSEE. **(1)** Chlorogenic acid, **(2)** Catechin, **(3)** 4-Hydroxybenzoic acid, **(4)** Orcinol, **(5)** 3-Hydroxybenzoic acid, **(6)** Naringin, **(7)** Salicylic acid, **(8)** Quercetin 3-*O*-β-D-glucoside, **(9)** Quercetin, **(10)** Chalcone, **(11)** Succinic acid, **(12)** 2-hydroxycinnamic acid, **(13)** Phloridzin dihydrate, **(14)** Flavone.

Prediction	1	2	3	4	5	6	7	8	9	10	11	12	13	14
ADME Prediction Absorption Parameters														
Bioavailability score	0.11	0.55	0.85	0.55	0.85	0.17	0.17	0.85	0.55	0.55	0.85	0.85	0.17	0.55
Caco-2 Permeability	−0.84	−0.283	1.151	1.677	1.123	−0.658	1.151	0.242	−0.229	1.335	0.603	1.210	0.203	1.263
Intestinal Absorption (%)	36.37	68.82	83.96	91.78	79.08	25.79	83.88	47.99	77.20	94.97	71.74	93.49	28.00	97.38
Distribution Parameters														
Log K*_p_* (cm/s)	−2.735	−2.735	−2.723	−2.585	−2.735	−2.735	−2.723	−2.735	−2.735	−1.998	−2.735	−2.712	−2.735	−2.215
VDss	0.581	1.027	−1.557	0.134	−1.607	0.619	−1.570	1.846	1.559	0.365	−1.013	−1.191	0.596	0.129
BBB Permeability	−1.407	−1.054	−0.334	−0.292	−0.397	−1.600	−0.334	−1.688	−1.098	0.560	−0.163	−0.225	−1.146	0.165
Metabolism Parameters														
CYP2D6, and CYP3A4 Substrate	No	No	No	No	No	No	No	No	No	No	No	No	No	No
CYP2D6, and CYP3A4 Inhibitors	No	No	No	No	No	No	No	No	No	No	No	No	No	No
Excretion Parameters														
Total Clearance	0.307	0.183	0.593	0.552	0.588	0.318	0.607	0.394	0.407	0.223	0.722	0.736	0.258	0.382
Renal OCT2 Substrate	No	No	No	No	No	No	No	No	No	No	No	No	No	No

**BBB:** blood-brain barrier; Log BB > 0.3, molecule BBB permeant, Log BB < −1 molecule poorly distributed across the BBB.

**Table 5:** PASS prediction of the major compounds found in CSEE.

Compounds	Biological Activities							
	Antioxidant		Antibacterial		Antifungal		Antineoplastic (Breast Cancer)	
	P_a_	P_i_	P_a_	P_i_	P_a_	P_i_	P_a_	P_i_
**Chlorogenic acid**	0.785	0.004	0.537	0.013	0.638	0.014	0.391	0.033
**Catechin**	**0.810**	**0.003**	0.320	0.053	0.552	0.023	0.486	0.020
**4-Hydroxybenzoic acid**	0.320	0.020	0.384	0.034	0.384	0.053	0.168	0.118
**Orcinol**	0.440	0.009	0.325	0.051	0.416	0.047	0.368	0.038
**3-Hydroxybenzoic acid**	0.329	0.019	0.373	0.037	0.378	0.055	0.187	0.103
**Naringin**	**0.851**	**0.003**	0.669	0.005	**0.816**	**0.004**	**0.858**	**0.006**
**Salicylic acid**	0.318	0.020	0.404	0.029	0.395	0.051	n.d.	n.d.
**Quercetin 3-O-β-D-glucoside**	**0.913**	**0.003**	0.599	0.009	**0.714**	**0.009**	**0.833**	**0.008**
**Quercetin**	**0.872**	**0.003**	0.387	0.033	0.490	0.032	**0.797**	**0.012**
**Chalcone**	0.421	0.010	0.284	0.066	0.361	0.059	0.544	0.015
**Succinic acid**	0.251	0.036	0.288	0.065	0.343	0.065	n.d.	n.d.
**2-hydroxycinnamic acid**	0.523	0.006	0.355	0.042	0.464	0.037	0.352	0.041
**Phloridzin dihydrate**	0.655	0.004	0.551	0.012	0.651	0.013	0.606	0.044
**Flavone**	0.469	0.008	0.286	0.065	0.369	0.057	0.597	0.010

Pa, probability ‘to be active’; Pi, probability ‘to be inactive’. Bold number: indicate a probable activity > 0.70.

**Table 6:** Prediction of toxicity, and the toxic endpoints of the major compounds found in CSEE. **(1)** Chlorogenic acid, **(2)** Catechin, **(3)** 4-Hydroxybenzoic acid, **(4)** Orcinol, **(5)** 3-Hydroxybenzoic acid, **(6)** Naringin, **(7)** Salicylic acid, **(8)** Quercetin 3-*O*-β-D-glucoside, **(9)** Quercetin, **(10)** Chalcone, **(11)** Succinic acid, **(12)** 2-hydroxycinnamic acid, **(13)** Phloridzin dihydrate, **(14)** Flavone. * Predi.: Prediction; **: Prob.: Probability.

N	Predicted LD_50_ (mg/kg)	Class	Hepatotoxicity		Carcinogenicity		Immunotoxicity		Mutagenicity		Cytotoxicity	
			Predi. *	Prob. **	Predi.	Prob.	Predi.	Prob.	Predi.	Prob.	Predi.	Prob.
**1**	5000	V	Ina.	0.72	Ina.	0.68	**Act.**	**0.99**	Ina.	0.93	Ina.	0.80
**2**	10,000	VI	Ina.	0.72	Ina.	0.51	Ina.	0.96	Ina.	0.55	Ina.	0.84
**3**	2200	V	Ina.	0.52	Ina.	0.51	Ina.	0.99	Ina.	0.99	Ina.	0.86
**4**	770	IV	Ina.	0.81	Ina.	0.72	Ina.	0.99	Ina.	0.98	Ina.	0.90
**5**	2000	IV	Ina.	0.52	Ina.	0.51	Ina.	0.99	Ina.	0.99	Ina.	0.86
**6**	2300	V	Ina.	0.81	Ina.	0.90	**Act.**	**0.99**	Ina.	0.73	Ina.	0.66
**7**	2300	V	Ina.	0.81	Ina.	0.90	**Act.**	**0.99**	Ina.	0.73	Ina.	0.66
**8**	1034	IV	**Act.**	**0.51**	Ina.	0.67	Ina.	0.99	Ina.	0.98	Ina.	0.86
**9**	159	III	Ina.	0.69	**Act.**	**0.68**	Ina.	0.87	Ina.	0.51	Ina.	0.99
**10**	1048	IV	Ina.	0.68	Ina.	0.64	Ina.	0.78	Ina.	0.99	Ina.	0.98
**11**	2260	V	Ina.	0.83	Ina.	0.80	Ina.	0.99	Ina.	0.98	Ina.	0.75
**12**	2850	V	**Act.**	**0.53**	**Act.**	**0.51**	Ina.	0.86	Ina.	0.92	Ina.	0.81
**13**	3000	V	Ina.	0.83	Ina.	0.82	Ina.	0.83	Ina.	0.85	Ina.	0.84
**14**	2500	V	Ina.	0.70	**Act.**	**0.69**	Ina.	0.99	Ina.	0.54	**Act.**	**0.75**

**GHS hazard classes: III:** 50 mg/kg < LD50 < 300 mg/kg, toxic if swallowed; **IV:** 300 mg/kg < LD50 ≤ 2000 mg/kg, harmful if swallowed; **V:** 2000 mg/kg < LD50 ≤ 5000 mg/kg, may be harmful if swallowed; **VI:** LD50 > 5000 mg/kg, non-toxic compounds.

**Table 7:** Free radical scavenging and antioxidant capacity of *C. siliqua* ethanolic extract (CSEE). The data is presented as the mean ± standard error of the mean (SEM) with a sample size of n = 3.

Extract/Reference	DPPH Scavenging Capacity IC_50_ (µg/mL)	β-Carotene Bleaching Assay (µg/mL)	ABTS Scavenging (TE µmol/mL)	Total Antioxidant Capacity *
CSEE	302.78 ± 7.55	352.06 ± 12.16	48.13 ± 3.66	165 ± 7.66
Ascorbic acid (AA)	260.24 ± 6.45	-	8.23 ± 0.97	-
Butylated hydroxytoluene (BHT)	-	29.23 ± 9.34	-	-

* Total antioxidant capacity reported as µg ascorbic acid equivalents per milligram of extract. **TE:** Trolox equivalent.

**Table 8:** Results of the antibacterial activities of *C. siliqua* Ethanolic Extract (CSEE).

Bacterial Strains	Gram Type	CSEE				Imipeneme (10 µg/disc)	Amoxicillin (25 µg/disc)
		IZ * (mm)	MIC (µL/mL)	MBC (µL/mL)	MBC/MIC	IZ (mm)	IZ (mm)
*S. aureus*	G+	21 ± 1.50	0.35	0.70	2	19 ± 0.50	13 ± 1.30
*E. faecalis*	G+	24 ± 0.66	0.35	0.35	1	15 ± 0.33	11 ± 0.66
*E. coli*	G-	28 ± 0.33	0.35	0.35	1	19 ± 0.66	18 ± 0.33
*E. vekanda*	G-	23 ± 0.50	0.35	0.35	1	24 ± 0.66	9 ± 1.5
*P. aeruginosa*	G-	18 ± 0.66	0.35	0.70	2	23 ± 0.33	16 ± 0.33

* **IZ:** Inhibition zone; **MIC:** Minimum Inhibitory Concentration; **MBC:** Minimum Bactericidal Concentration; *S. aureus* ATCC 29213, *E. faecalis* ATCC 29212, *E. coli* ATCC 25922, *P. aeruginosa* ATCC 29212.

**Table 9:** Results of the antifungal activities of *C. siliqua* Ethanolic Extract (CSEE).

Fungal Strains	CSEE				Cycloheximide (1 mg/mL)
	IZ (mm)	MIC (µL/mL)	MFC (µL/mL)	MFC/MIC	IZ (mm)
*C. albicans*	24 ± 1.00	10	10	1	22 ± 0.66
*G. candidum*	23 ± 0.66	10	10	1	18 ± 0.5

**IZ:** Inhibition zone; **MIC:** Minimum Inhibitory Concentration; **MFC:** Minimum Fungicidal Concentration.

**Table 10:** Assessment of the selectivity indexes and IC_50_ levels for the *C. siliqua* ethanolic extract (CSEE) on various human breast cancer cell lines.

Treatments	IC_50_ Value ± SD (µg/mL) *				Selectivity Index (SI)		
	MCF-7	MDA-MB-231	MDA-MB-436	PBMC	MCF-7	MDA-MB-231	MDA-MB-436
**CSEE**	32.44 ± 5.23	40.05 ± 3.21	53.55 ± 5.35	891.30 ± 28.10	**27.47**	**22.25**	**16.64**
**Cisplatin**	5.19 ± 1.85	4.40 ± 1.20	6.73 ± 1.33	32.88 ± 5.28	**6.33**	**7.47**	**4.88**

* The mean values of three separate experiments were calculated and presented as means with standard deviations. The selectivity index was calculated as the ratio of the IC_50_ values of PBMC and tumor cells.

## Data Availability

Data is contained within the article.
